# Supplementary material for: The Role of Viral Interference in Shaping RSV Epidemics Following the 2009 H1N1 Influenza Pandemic
Source: Influenza Other Respir Viruses. 2025 Apr 25;19(4):e70111. doi: 10.1111/irv.70111 (PMC12022500; doi:10.1111/irv.70111)
Supplement: Supplementary file 1 — Figure S1 Laboratory‐confirmed positive tests for the pdmH1N1 virus and RSV following the pandemic. Region 2 (NJ: New Jersey, NY: New York, PR: Puerto Rico, VI: the Virgin Islands); Region 3 (DE: Delaware, DC: District of Columbia, MD: Maryland, PA: Pennsylvania, VA: Virginia, WV: West Virginia); Region 5 (IL: Illinois, IN: Indiana, MI: Michigan, MN: Minnesota, OH: Ohio, WI: Wisconsin); Region 6 (AR: Arkansas, LA: Louisiana, NM: New Mexico, OK: Oklahoma, TX: Texas); Region 7 (IA: Iowa, KS: Kansas, MO: Missouri, NE: Nebraska); Region 9 (AZ: Arizona, CA: California, HI: Hawaii, NV: Nevada). (A) shows positive laboratory tests for pdmH1N1 virus (red) and RSV (black) before (dashed line), during (shaded area) and after the 2009 influenza pandemic in the six regions of the US. Dashed grey lines indicate 1st January of the year. (B) shows RSV activity following the pandemic and in other normal epidemic seasons. RSV activity during the pandemic 2009/10 season is highlighted in the black box. Figure S2 Optimal alignments based on the DTW computational method. An optimal alignment shows the indices of the elements in the query time‐series that correspond to those in the reference time series (e.g., RSV time‐series in 2009/10 and 2010/11 seasons). If the path crosses below the diagonal line, it indicates that the query time‐series lags behind the reference time‐series, while crossing above suggests a lead. If the path is perfectly aligned on the diagonal line, it shows no difference. Some conditions on the warping curve were used to generate meaningful outcomes. A 35‐week window (a Sakoechiba window) was posed to prevent excessively long lead/lag times. An asymmetric step pattern with a P2 slope constraint was used to facilitate a leading alignment between the time series. The computed optimal alignment paths for RSV activity during the 2009/10 and 2010/11 seasons with respect to other seasons in different regions of the US are given in blue and purple, respectively. Figure S [file IRV-19-e70111-s001.docx]

**Supplementary Materials**

**The role of viral interference in shaping RSV epidemics following the 2009 H1N1 influenza pandemic**

Ke Li^1^*, Deus Thindwa^1^, Daniel M Weinberger^1^, Virginia E Pitzer^1^

^1^Department of Epidemiology of Microbial Diseases, Yale School of Public Health, New Haven, CT, USA.

*Corresponding authors: [ke.li.kl662@yale.edu](mailto:ke.li.kl662@yale.edu)

**Dynamic time warping and hierarchical clustering**

We used a dynamic time warping (DTW) to calculate the pairwise non-linear alignment of the 12 RSV time-series (corresponding to the 12 seasons) in each region of the US (**SFig. 2**) and quantify dissimilarity between those time-series. The DTW algorithm computed the optimum warping path between two series under certain constraints, including monotonicity, continuity, warping window, and boundary. The R packages *dtw* and dtwclust facilitated the implementation of the algorithm and optimization [4-5]. After calculating distances, a local cost matrix (*lcm*) was generated with dimensions of $n \times m$, where $n$ and $m$ represent the lengths of the pairwise time series. Considering the input time series $Q$ and $S$, for each element $(i,j)$ of the lcm, the distance between $Q_{i}$ and $R_{j}$ was computed, such that $lcm(i,j)=(\sum|Q_{i}-R_{j}|^{p})^{1/p}$. The DTW algorithm thereby identifies the path that minimizes the alignment between pairwise time-series $Q$ and $R$ by iteratively stepping through the lcm, starting at *lcm*$(1,1)$ and finishing at lcm$(n,m)$, while aggregating the cost. At each step, the algorithm determines the direction in which the cost increases the least under the given constraints.

To limit the area of the lcm that the DTW algorithm must traverse, we implemented the Sakoe-Chiba window as a global constraint [5]. This constraint confines the allowed region along the diagonal of the lcm. To select an optimal window size for hierarchical clustering, we evaluated clustering using the modified Davies-Bouldin (DB) internal cluster validity index (CVI), iterating across different values of window size from 1 to 52, corresponding to 1 to 52 weeks, while keeping cluster size as 3. For each window size, the DB CVI calculated distances from computed cluster centroids.

We subsequently performed hierarchical clustering on the distances of aligned time-series using DTW. We utilized the Ward D2 clustering method, which minimizes the sum of squared differences from the centroid during the merging of clusters. This hierarchical clustering of the 12 RSV epidemics in each region created a hierarchy of groups. As the level in the hierarchy increased, clusters were formed by merging clusters from the next lower level, resulting in an ordered sequence of groupings.

**The center of gravity, and intensity of RSV activity**

The center of gravity of RSV activity for each season in each region ($G_{s,r}$) was measured as the mean epidemic week, with each week weighted by the number of positive tests, such that $G_{s,r}=\sum_{w\in[1:52]} w \times Y_{s,r,w}/ \sum_{w\in[1:52]} Y_{s,r,w}$, where $w$ is an index for the week of each epidemic year, and $Y_{s,r,w}$ is the number of rescaled positive RSV tests in region $r$ during epidemic season $s$ and week $w$. To determine the RSV intensity for each season and region, we calculated the area under the curve using *trapz* function in R (version 4.0.2).

**Transmission dynamic models**

We used an age-stratified Susceptible-Infected-Susceptible (SIS) model, taking into account repeat infections, to describe the transmission dynamics of RSV. The model was initially proposed by Pitzer et al. [1] to study the environmental drivers of the spatiotemporal dynamics of RSV in the US. The model assumed individuals were born with protective maternal immunity, which waned exponentially, leaving the infants susceptible to infection. We assumed a progressive build-up of immunity following up to four previous infections. Following infection with RSV, individuals developed partial immunity, reducing the rate of subsequent infection and relative infectiousness of the following infections. We also assumed subsequent infections had a shortened recovery time compared to primary infections. Transmission-relevant contact patterns were assumed to be frequency-dependent and were consistent with the previous work [1]. The model was able to reproduce the seasonal annual or biennial patterns of RSV transmission in different regions of the US. To model influenza transmission dynamics, we used a Susceptible-Infected-Recovered-Susceptible (SIRS) model. We assumed waning immunity for recovered individuals, allowing influenza infection to recur following the influenza pandemic in the 2009/10 and 2010/11 seasons.

The RSV and influenza transmission models were coupled through three hypothetical viral interference mechanisms. The first mechanism assumed influenza infection reduced the host's susceptibility to subsequent RSV coinfections, modulating the infection rate of susceptible individuals (**SFig. 4A**), captured by a parameter $\theta$, i.e. $dX_{si}/dt=-\theta\lambda_{1}X_{si}+\lambda_{2}X_{ss}-\gamma_{2}X_{si}$ (**Eq. (10)**). The second mechanism assumed influenza infection reduced the infectious period (i.e. increased the rate of recovery) of subsequent RSV coinfections (**SFig. 4B**), captured by a parameter $\eta$, i.e. $dX_{i1i}/dt=-\eta\gamma_{1}X_{ii}+\lambda_{1}X_{si}+\lambda_{2}X_{is}-\gamma_{2}X_{ii}$ (**Eq. (11)**). The third mechanism assumed influenza infection reduced the force of infection of RSV (**SFig. 4C**), captured by a parameter $\xi$, i.e. $\lambda_{1}=\xi\beta_{1}(t)X_{ai}$, where $X_{ai, a = \{i1,i2,i3,i4\}}$ represents coinfection terms. The model was described by a system of ordinary differential equations.

**Model calibration**

To calibrate the model parameters, we first fit the RSV dynamic model to the laboratory reports of positive RSV specimens from 2007 to 2019. We estimated the baseline transmission rate ($\beta_{1}$), seasonal amplitude ($\alpha_{1}$), seasonal offset ($\phi_{1}$) and reporting fraction ($f$) for each region, respectively, using maximum likelihood estimation. The likelihood of the data given the model was calculated by assuming the number of positive cases in each week was Poisson-distributed with a mean equal to the model-predicted cases times the reporting fraction. Other parameter values of the model were adopted from [1] and are provided in **Table 1** below. For the influenza model, we assumed the mean infectious period for primary and secondary influenza infections is 8 days, the duration of waning immunity is 40 weeks [7,8]. and the seasonal amplitude is equal to that estimated for RSV. We started by simulating only the RSV epidemic model, seeding the model with one RSV-infected individual in each age group except the <1 year-old age group. We used a burn-in period of 60 or 61 years, depending upon the region that exhibits either an annual or biennial RSV pattern, to ensure the RSV model reached an equilibrium quasi-steady state. Influenza infection was introduced to the population after the model reached the equilibrium quasi-steady state.

To estimate the effects of viral interference from pdmH1N1 infection on shaping RSV epidemics, we applied Latin Hypercube Sampling (LHS) to generate representative samples from a wide range of values for the parameter space $\Phi=(\Theta, \beta_{2}, \phi_{2}, \tau)$, where $\Theta=(\theta, \xi, \eta)$ represents interference parameters; $\beta_{2}$ is the transmission rate of the pdmH1N1 virus; $\phi_{2}$ is the seasonal phase offset of influenza dynamics, and $\tau$ is the time point when influenza infection is seeded in the population. We generated 100,000 samples from a uniform distribution $U(0,1)$ for the parameter $\theta$ and $\xi$, respectively, and from a uniform distribution $U(1,3)$ for the parameter $\eta$. We also sampled 100,000 values from a uniform distribution $U(100,110)$ for the parameter $\tau$, mimicking the onset time of the second H1N1 pandemic wave during the winter of 2009. Additionally, 100,000 samples were obtained from a uniform distribution $U(2,3)$ for the parameter $\beta_{2}$, based on the estimates of the basic reproduction number of the H1N1 pandemic virus in the United States. 100,000 values of seasonal offset of influenza $\phi_{2}$ were sampled from a uniform distribution $U(-1,1).$

Then, we explored the parameter space for each viral interference mechanism separately. We generated forward simulations using the sampled parameter sets and fitted them to weekly RSV admissions from July 2008 to June 2018. We calculated the log-likelihoods of the model under each parameter set, assuming the number of hospitalizations in each age class during each week was Poisson-distributed with a mean equal to the model-predicted number times the estimated reporting fraction. We then normalized the log-likelihoods (as weights) of each parameter set and resampled 10,000 parameters from the joint distribution based on the weights. The data that we used was the weekly RSV positive tests in the 2009/10 season in Region 1 (CT: Connecticut, ME: Maine, MA: Massachusetts, NH: New Hampshire, RI: Rhode Island, and VT: Vermont), Region 4 (AL: Alabama, FL: Florida, GA: Georgia, KY: Kentucky, MS: Mississippi, NC: North Carolina, SC: South Carolina, and TN: Tennessee) or Region 10 (AK: Alaska, ID: Idaho, OR: Oregon, and WA: Washington), respectively.

**Supplementary Figures**

**Supplements Figure 1. Laboratory-confirmed positive tests for the pdmH1N1 virus and RSV following the pandemic**. Region 2 (NJ: New Jersey, NY: New York, PR: Puerto Rico, VI: the Virgin Islands); Region 3 (DE: Delaware, DC: District of Columbia, MD: Maryland, PA: Pennsylvania, VA: Virginia, WV: West Virginia); Region 5 (IL: Illinois, IN: Indiana, MI: Michigan, MN: Minnesota, OH: Ohio, WI: Wisconsin); Region 6 (AR: Arkansas, LA: Louisiana, NM: New Mexico, OK: Oklahoma, TX: Texas); Region 7 (IA: Iowa, KS: Kansas, MO: Missouri, NE: Nebraska); Region 9 (AZ: Arizona, CA: California, HI: Hawaii, NV: Nevada). (A) shows positive laboratory tests for pdmH1N1 virus (red) and RSV (black) before (dashed line), during (shaded area) and after the 2009 influenza pandemic in the six regions of the US. Dashed grey lines indicate 1st January of the year. (B) shows RSV activity following the pandemic and in other normal epidemic seasons. RSV activity during the pandemic 2009/10 season is highlighted in the black box.

**Supplements Figure 2. Optimal alignments based on the DTW computational method**. An optimal alignment shows the indices of the elements in the query time-series that correspond to those in the reference time series (e.g., RSV time-series in 2009/10 and 2010/11 seasons). If the path crosses below the diagonal line, it indicates that the query time-series lags behind the reference time-series, while crossing above suggests a lead. If the path is perfectly aligned on the diagonal line, it shows no difference. Some conditions on the warping curve were used to generate meaningful outcomes. A 35-week window (a Sakoechiba window) was posed to prevent excessively long lead/lag times. An asymmetric step pattern with a P2 slope constraint was used to facilitate a leading alignment between the time series. The computed optimal alignment paths for RSV activity during the 2009/10 and 2010/11 seasons with respect to other seasons in different regions of the US are given in blue and purple, respectively.


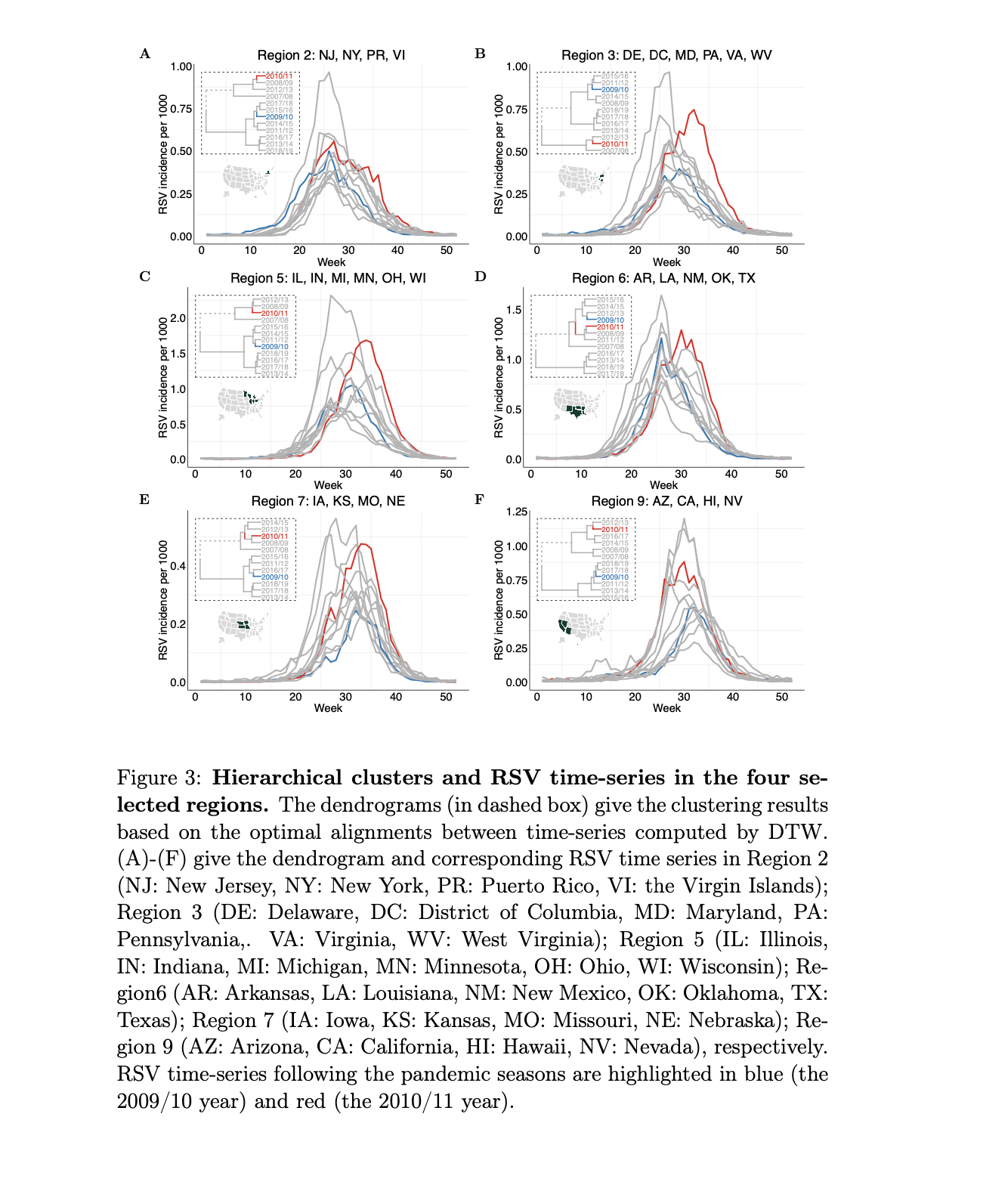
**Supplements Figure 3. Hierarchical clusters and RSV time-series in the six selected regions.** The dendrograms (in dashed box) give the clustering results based on the optimal alignments between time-series computed by DTW. (A)-(F) give the dendrogram and corresponding RSV time series in Region 2 (NJ: New Jersey, NY: New York, PR: Puerto Rico, VI: the Virgin Islands); Region 3 (DE: Delaware, DC: District of Columbia, MD: Maryland, PA: Pennsylvania, VA: Virginia, WV: West Virginia); Region 5 (IL: Illinois, IN: Indiana, MI: Michigan, MN: Minnesota, OH: Ohio, WI: Wisconsin); Region6 (AR: Arkansas, LA: Louisiana, NM: New Mexico, OK: Oklahoma, TX: Texas); Region 7 (IA: Iowa, KS: Kansas, MO: Missouri, NE: Nebraska); Region 9 (AZ: Arizona, CA: California, HI: Hawaii, NV: Nevada), respectively. RSV time-series following the pandemic seasons are highlighted in blue (the 2009/10 year) and red (the 2010/11 year).

**Supplements Figure 4. Proposed viral interference mechanisms and model diagrams.** Three viral interference mechanisms from influenza on RSV transmission were proposed: the hosts infected with pdmH1N1 enable: (A) the reduction of the host's susceptibility to following RSV infection by a proportion $\theta$,(B) the reduction of the infectious period of RSV infection by a proportion $\eta$, and (C) the reduction of the force (i.e., $\lambda_{1}$) of infection of RSV by a proportion $\xi$.

**Supplements Figure 5. Predictions of RSV with viral interference mechanisms.** Model predictions of RSV epidemics in Region 10 where RSV activity exhibits a biennial pattern. In the baseline model, we assume no viral interference effects (i.e., $\theta=1$, $\eta=1$or $\xi=1$). In the model with viral interference, we use median estimates of (A) $\eta$ or (B) $\xi$, and set the other two effects to the baseline values.


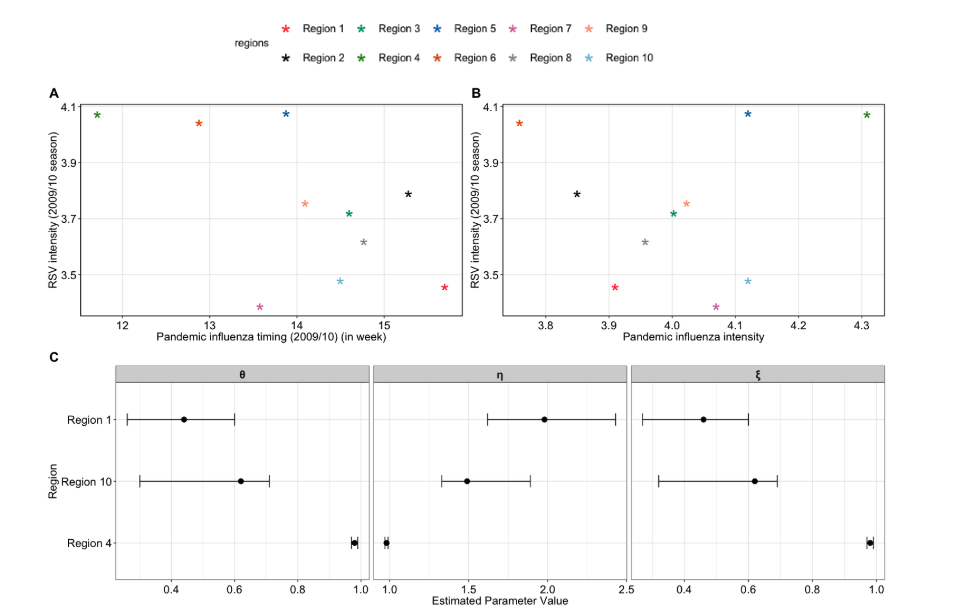


**Supplemental Figure 6 The activity of the H1N1 influenza pandemic and the estimated viral interference parameters**. (A) The scatter plot between RSV intensity during the 2009/10 pandemic season and the mean timing (measured by the center of gravity) of the second wave of the pandemic influenza. (B) The correlation between reduced RSV activity during the 2009/10 season and the pandemic influenza between April 2009 and June 2010. (C) Parameter estimates of viral interference for selected regions.


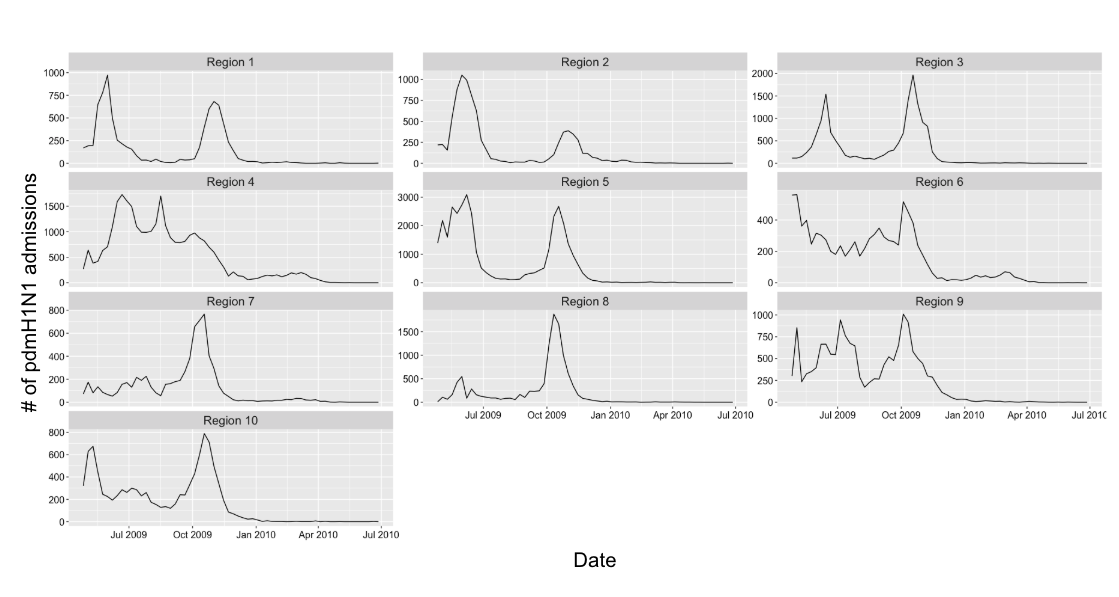


**Supplemental Figure 7 Time series of the H1N1 influenza pandemic in 10 regions in the United States.**

**References**

1. V. E. Pitzer, C. Viboud, W. J. Alonso, T. Wilcox, C. J. Metcalf, C. A. Steiner, A. K. Haynes, B. T. Grenfell, Environmental drivers of the spatiotemporal dynamics of respiratory syncytial virus in the united states, PLoS pathogens 11 (1) (2015) e1004591.
2. Z. Zheng, V. E. Pitzer, E. D. Shapiro, L. J. Bont, D. M. Weinberger, Estimation of the timing and intensity of reemergence of respiratory syncytial virus following the covid-19 pandemic in the us, JAMA network open 4 (12) (2021) e2141779–e2141779.
3. G. De Serres, I. Rouleau, M.-E. Hamelin, C. Quach, D. Skowronski, L. Flamand, N. Boulianne, Y. Li, J. Carbonneau, A.-M. Bourgault, et al., Contagious period for pandemic (H1N1) 2009, Emerging infectious diseases 16 (5) (2010) 783.
4. [Senin P. Dynamic time warping algorithm review. 2008. Available:](http://paperpile.com/b/ErJw75/uJvi) <https://www.semanticscholar.org/paper/Dynamic-Time-Warping-Algorithm-Review-Senin/8096fe77e22ee54d829861642ac71064d866b913>
5. [Giorgino T. Computing and Visualizing Dynamic Time Warping Alignments in R: The dtw Package. J Stat Softw. 2009;31: 1–24.](http://paperpile.com/b/ErJw75/cD7Q)
6. [White LF, Wallinga J, Finelli L, Reed C, Riley S, Lipsitch M, et al. Estimation of the reproductive number and the serial interval in early phase of the 2009 influenza A/H1N1 pandemic in the USA. Influenza Other Respi Viruses. 2009;3: 267–276.](http://paperpile.com/b/ErJw75/QKd6)

7. [Baker RE, Park SW, Yang W, Vecchi GA, Metcalf CJE, Grenfell BT. The impact of COVID-19 nonpharmaceutical interventions on the future dynamics of endemic infections. Proceedings of the National Academy of Sciences. 2020;117: 30547–30553.](http://paperpile.com/b/ls4GUV/UzPG)

8. [Baker RE, Saad-Roy CM, Park SW, Farrar J, Metcalf CJE, Grenfell BT. Long-term benefits of nonpharmaceutical interventions for endemic infections are shaped by respiratory pathogen dynamics. Proc Natl Acad Sci U S A. 2022;119: e2208895119.](http://paperpile.com/b/ls4GUV/dk3Q)
